# Taming the endless scroll? Short-form videos, digital routines and neurocognitive outcomes in youth: a systematic literature review

**DOI:** 10.1007/s00787-026-03083-7

**Published:** 2026-06-13

**Authors:** Marlene Ebster, Michael Lauerer, Eckhard Nagel, Sebastian Schmidt

**Affiliations:** 1 Institute of Management for Medicine and Health Science, University of Bayreuth, Prieserstr. 2, 95444 Bayreuth, Bavaria Germany; 2 School of Computer Science, Queensland University of Technology, 2 George St, Brisbane, 4000 Queensland Australia; 3 The Medical University of Lausitz – Carl Thiem, Thiemstr. 111, 03048 Cottbus, Brandenburg Germany

**Keywords:** Short-form video, TikTok, Development, Social media, Cognition, Flourishing, Digital health

## Abstract

Short-form videos (SFVs) deliver digital content in a way that sets them apart from other media. Concerns have arisen that such user interfaces may diminish attention, memory and emotional wellbeing in young people, yet evidence has not previously been synthesised for this demographic and in isolation from other media types. To assess the association between exposure to SFV-interfaces and neurocognitive outcomes in individuals aged $\boldsymbol{\le 25}$ years, and to appraise the certainty of this evidence. Following PRISMA and Cochrane guidelines, four databases (PubMed, PsycINFO, WoS, Cochrane Reviews) were searched (2015–2025). Of 1,498 records, 42 studies (N = 46,912; mean age = 16.8) met the PICOS criteria. Most were cross-sectional (88%), with two longitudinal, one EEG, and one MRI study. Risk of bias and quality were evaluated using GRADE. Heavy, unstructured SFV use (typically $\boldsymbol{\ge 4}$ h/day) was consistently associated with: small-to-moderate increases in inattention ($\boldsymbol{\beta \approx 0.30}$) and impulsivity ($\boldsymbol{\beta \approx 0.25}$); moderate reductions in working-memory span ($\boldsymbol{\beta \approx -0.75}$) and self-regulation ($\boldsymbol{\beta \approx -0.45}$); moderate elevations in anxiety ($\boldsymbol{\beta \approx 0.35}$), depression ($\boldsymbol{\beta \approx 0.48}$) and stress ($\boldsymbol{\beta \approx 0.32}$); and a moderate-to-large surge in addiction symptoms ($\boldsymbol{\beta \approx 0.60}$). Two cross-sectional imaging studies also reported associations between heavier short-video use and neurobiological measures. These included grey-matter volume differences in the orbitofrontal cortex and cerebellum, altered resting-state signal synchrony, and reduced P300 amplitudes. Protective factors included supportive environments, digital routines, and digital literacy. Consistent, albeit preliminary, evidence links excessive SFV watching to cognitive and emotional challenges in youth. Longitudinal and experimental studies are urgently needed, alongside trials of modifiable levers to reduce addiction-like use and related harms.

## Introduction

Short-form videos on platforms such as TikTok, Instagram Reels, and YouTube Shorts have become a constant part of the developmental environment of many children and adolescents. In 2023, more than a quarter of TikTok’s users were aged 13–17 years [1], and, despite age restrictions, millions of U.S. children under 13 accessed these apps in 2022 [2]. This reach implies substantial, often daily exposure across paediatric and adolescent populations.

Yet, prevailing clinical guidance and much of the empirical literature still treat ”screen time” or ”social media” as essentially homogeneous exposures. That assumption is increasingly difficult to defend. The way short-form videos (SFVs) are delivered – pace, algorithmic curation, and infinite scroll [3, 4] – differs materially from both television and legacy social networks. Preclinical work indicates that early-life audiovisual overstimulation can reshape behaviour and brain structure [5–8]. These findings underscore the biological plausibility of effects during two developmental windows (childhood and adolescence) when attention control, reward prediction, and self-regulation are still maturing.

Current syntheses, however, often conflate content, platform type and delivery design, which obscures risk stratification and blunts practice advice. TikTok, for example, has been described as a ”neuro-image” environment characterised by fragmented, affect-driven sequences [9], while empirical studies associate SFV viewing with flow states, self-control failures, and addictive use patterns [10, 11]. Moreover, screen dependency in younger children has been linked to impulsivity, inattention, anxiety, and learning difficulties [12, 13]. Nonetheless, influential reviews still emphasise television or gaming [14–16], only a few consider media pace [17, 18], and large longitudinal analyses often operationalise aggregated screen time across heterogeneous platforms [19]. Although their work also foregrounds design features such as endless scrolling and personalised feeds, it does not organise the evidence within a biopsychosocial framework or translate the findings into developmentally sensitive, modifiable levers for intervention and prevention. Consequently, clinicians and app designers continue to lack a structured account of which mechanisms may be amenable to change, and at which developmental stages such intervention points may be most relevant.

In this review, we treat the *user interface* of short-form video platforms as the exposure of interest. We separate delivery mechanics into three observable components: (a) personalised algorithmic curation, (b) infinite scroll and (c) rapidity. This distinction is used as a pragmatic analytic heuristic rather than as an exhaustive or causal taxonomy. Each component represents a distinguishable interface-level mechanism through which short-form video content is delivered. Existing work supports treating these features as analytically distinct. Rapidity captures the fast-paced transitions between short videos and the associated context switching, which has been linked to impaired prospective memory [20, 21]. Personalised algorithmic curation refers to the selection and sequencing of content for the user and has been discussed in relation to flow, time distortion, and problematic short-form video use [11]. Infinite scroll captures the continuity of access created by the removal of natural stopping cues, a design pattern that may prolong user sessions [22].

**Fig. 1:**
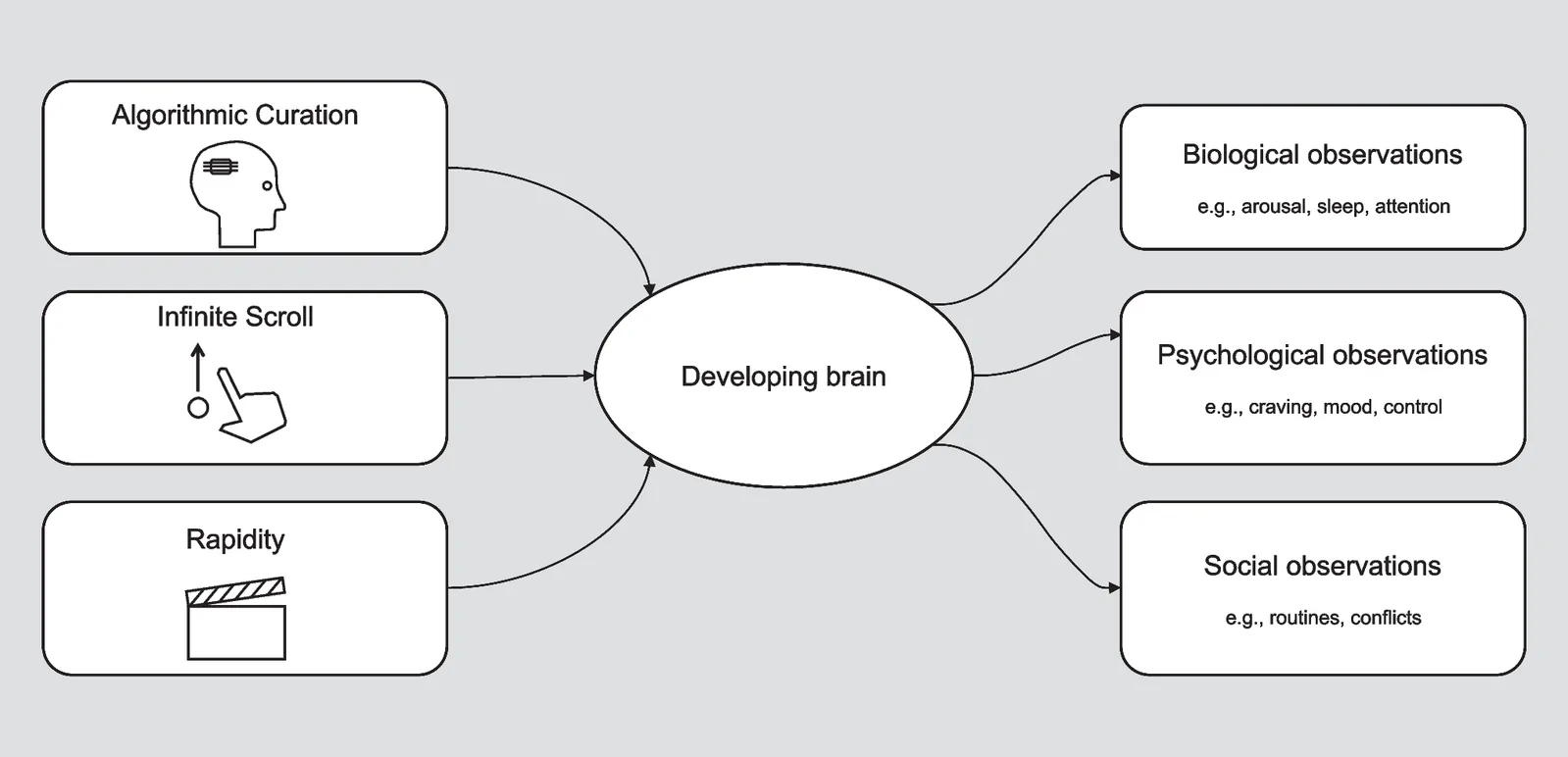
Conceptual overview of short-form video delivery mechanics, the developing brain and biopsychosocial observations. Arrows indicate hypothesised points of interaction and are intended as heuristic black boxes rather than established causal pathways

Importantly, two theoretical lenses guide the present review. Engel’s biopsychosocial model [23] provides a framework for conceptualising short-form video delivery mechanics as exposures that may shape adolescent outcomes through interacting biological, psychological, and social pathways. This framing is particularly relevant in childhood and adolescence, when reward processing, attention regulation, and self-regulation are still developing and may therefore be more responsive to highly salient digital environments. At the psychological level, the dual theory of habituation and sensitisation [24] helps explain how repeated exposure to rapidly changing, algorithmically curated content may alter reward sensitivity, attention, and engagement over time. Together, these theories provide a developmentally grounded rationale for examining not short-form video content in general, but the specific interface features through which it is delivered. To complement this, Fig. 1 offers a non-causal conceptual overview that juxtaposes these delivery mechanics with the developing brain and biological, psychological, and social observations. This framing separates delivery from content and provides a shared vocabulary for the subsequent synthesis.

The present review isolates the aforementioned three primary delivery mechanics and complements existing short-form video syntheses by shifting the focus from overall platform use to interface features. Importantly, we do not evaluate obviously harmful or inappropriate content (such as violent or drug-related videos). Instead, we ask whether ”the app’s design” *alone* is associated with adverse outcomes. Gathering data from toddlers to young adults up to 25 years, our objective is to clarify, confirm, or challenge current public and scientific concern while providing a more developmentally and mechanistically structured account than previous reviews. The upper age limit of 25 years reflects a pragmatic developmental boundary rather than a fixed maturational endpoint, given the heterogeneity of adolescent transitions and continued self-regulatory maturation into young adulthood [25, 26].

## Methods

### Search strategy and data sources

The review followed PRISMA guidance [27] and methodological standards from the Cochrane Handbook [28]. Two reviewers searched PubMed, PsycINFO, Web of Science and Cochrane Reviews in April 2025 for records published from January 2015 to April 2025 in English or German. The time window reflects the emergence of large-scale short-video platforms, including Musical.ly/Douyin (2014/2016), TikTok (2018 global launch), and the introduction of Instagram Reels and YouTube Shorts in 2020. We constructed a four-block Boolean query aligned with the Participants, Interventions, Comparisons, Outcomes (PICO) framework. Next, we iteratively refined terms using prior systematic reviews, which disclose their search strategies for related topics and keywords. The PubMed syntax is shown in Table 1. The full, database-specific syntax protocol (all four databases) is available in Supplementary material S1 on the Open Science Framework (OSF): https://osf.io/qks32/files/osfstorage/68d0341f49bfe7549f53d908. Where available, database filters were applied only for publication date and language. Population characteristics, including human participants and age range, were not applied uniformly as database filters because filter availability and indexing differed across databases. These criteria were therefore assessed manually during title/abstract and full-text screening according to the predefined eligibility criteria.

**Table 1:** Electronic search strategy

Selection level	Descriptors and Boolean operators
Population	(infants OR toddler OR preschoolers OR child* OR kids OR boys
	OR girls OR youth* OR teen* OR adolescen*) AND
short-video platforms	(tiktok OR douyin OR musical.ly OR vine OR youtube OR reels
	OR “social media app” OR “social media apps” OR “video platforms”
	OR “entertain* app*” OR short-videos OR “short-video*”
	OR “audiovisual stimulation” OR “visual stimulation”
	OR “social media feed” OR “fast video*” OR “fast pace* video*”
	OR “audiovisual content” OR “for you page” OR scroll*
	OR “algorithmically generated”OR “video recomm*”
	OR “rabbit hole”) AND
Mental-health comparators	(“reward system” OR mesocorticolimbic OR mesolimbic OR dopamin*
	OR serotonin* OR neuropsychiatric* OR “attention deficit*”
	OR anxiety OR depression OR cognitive OR “executive function”
	OR memory OR “attention problem*” OR inattention OR
	hyperactiv* OR “user habits” OR “inhibitory control”
	OR “novelty seeking” OR “harm avoidance” OR impulsivity
	OR “trait curiosity” OR “self regulat*” OR “emotional regulat*”
	OR “internet addiction” OR “social media addiction”
	OR “mobile phone addiction” OR “online social networking addiction”
	OR overstimulation OR gambling OR “flow experience”
	OR “academic performance” OR “learning motivation”
	OR “core self-evaluation” OR “self-concept clarity”
	OR “social belonging” OR “psychosocial functioning” OR friendships
	OR peers OR relationships OR “well being” OR wellbeing) AND
Outcome	(associat* OR “p-value” OR significant* OR effect OR impact
	OR link* OR mediat* OR moderat* OR scale OR questionnaire
	OR qol OR “quality of life”)

Pilot studies and pilot projects were excluded because the review aimed to synthesise empirical evidence from studies with completed designs and interpretable findings rather than feasibility-oriented or preliminary work. Test runs with abstract searches instead of full text in all four databases demonstrated a high match for the research question, confirming adequate sensitivity of the search string.

### Eligibility criteria and selection process

The eligibility criteria for this systematic review were established a priori to ensure that all included studies directly addressed the research objective using the Participants, Interventions, Comparisons, Outcomes, and Study Design framework [29]. This approach, consistent with PRISMA and Cochrane Handbook recommendations, was intended to maximize methodological rigor, ensure relevance, and minimize selection bias. The detailed inclusion and exclusion criteria are outlined in Table 2.

**Table 2:** Inclusion and exclusion criteria

Selection criteria	Inclusion criteria	Exclusion criteria
Study design	Systematic reviews, qualitative and quantitative studies	Study protocols, pilot studies, pilot projects, case studies, workshops
Publication form	Articles from specialist and scientific journals, final project reports	Working papers, news articles, bachelor’s and master’s theses, forums, dissertations, anthologies, duplicates, commentaries, monographs
Publication date	2015–2025	Published before 2015
Languages	English and German	Other languages
Availability	Free full text accessible	Only title/abstract accessible
Participants	Toddlers, preschoolers and older children, teenagers, adolescents up to 25 years old	Older than 25 years
Intervention	Platforms that host short-videos; video recommendations must be algorithmically generated based on user preferences	Predominantly messenger apps, television, (online) gaming platforms; studies on content quality on social media platforms
Comparators	Brain development indicators, mental health indicators, psychosocial indicators, behavioural changes, addictions	Physical health outcomes (e.g., back pain, eye health, abdominal pain); communicable disease prevention (e.g., COVID testing, portrayal of vaccine shots)
Outcome	Relationships and associations between short-video platforms and youth mental health	Theoretical descriptions only; development or validation of questionnaires and scales

**Fig. 2:**
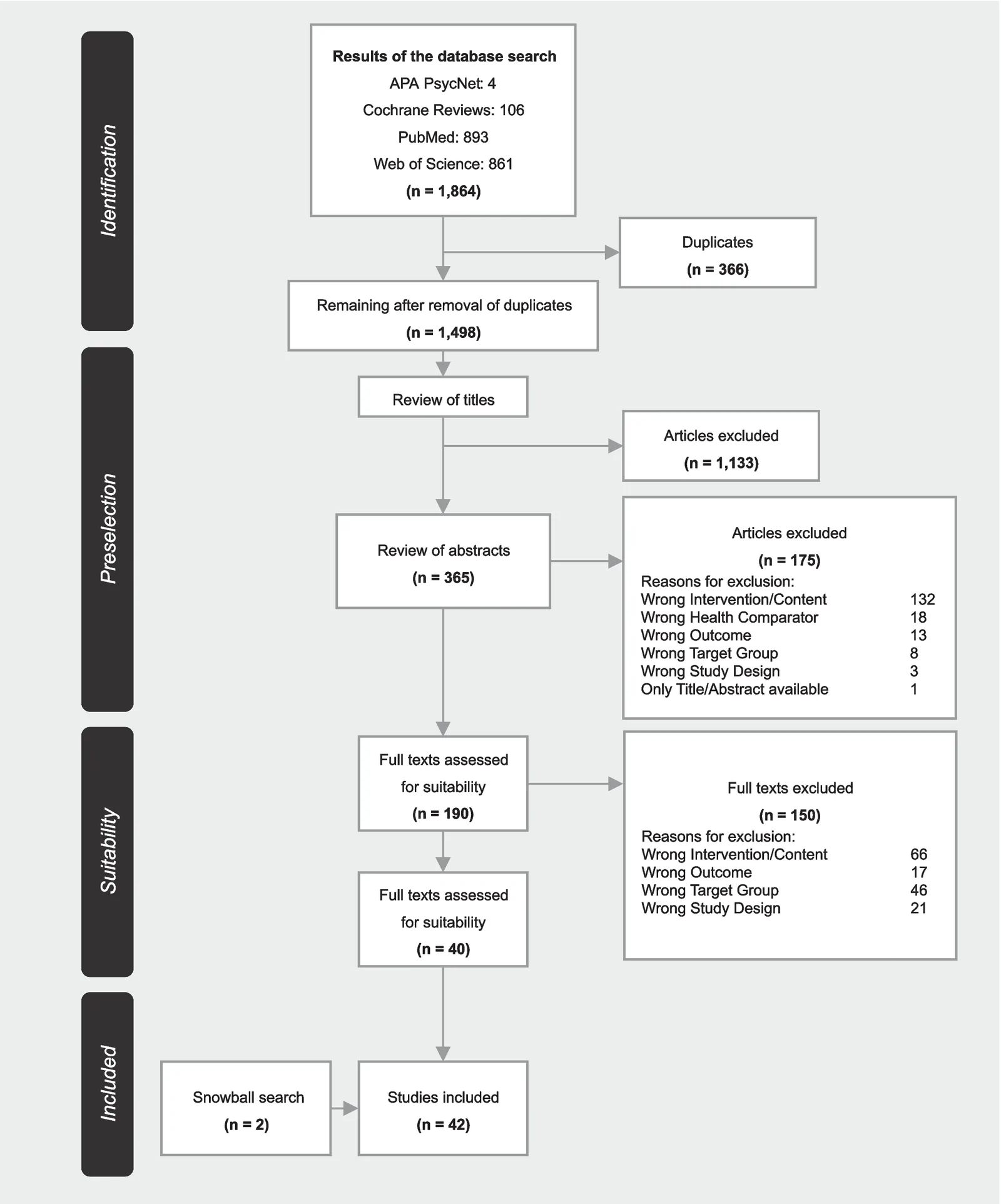
PRISMA-flow diagram

For the intervention criterion, “algorithmically generated” recommendations were defined as platform-mediated video streams that are automatically selected or sequenced for an individual user based on inferred or explicit user preferences. These preferences may be derived from prior viewing behaviour, likes, follows, searches, interactions, or broader engagement patterns. This criterion was used to distinguish personalised short-video feeds, such as TikTok, Douyin, Instagram Reels, and YouTube Shorts, from non-personalised video collections, manually curated playlists, television content, and messenger-based video sharing. For the present systematic review, a total of 1,864 records were initially identified across the four databases. The full process is illustrated as recommended by Moher et al. [30] in Fig. 2.

Reasons for exclusion are tabulated in Supplementary material S2, which was made available at the OSF under https://osf.io/qks32/files/osfstorage/684fc393567a560b45cc2c71. The selection process was carried out using blind-mode in Rayyan, allowing independent decisions of the two reviewers (M.E., S.S.) and ensuring systematic documentation and traceability throughout the selection process [31]. In all cases of disagreement, consensus was achieved through discussion.

Forty records from the database searches met the predefined eligibility criteria. We cross-referenced our penultimate set with studies in the identified review [32]. This produced 20 candidates. Two met our criteria and were added. Eighteen were excluded. The reasons are documented in Supplementary Material S3, which was made available at the OSF under: https://osf.io/qks32/files/osfstorage/684fc38ce0b46fc2a7b7880c. We did not perform additional forward or backward searches. Thus, the final corpus yields 42 studies.

### Quality assessment

To appraise methodological quality and overall certainty, we applied the GRADE (Grading of Recommendations Assessment, Development, and Evaluation) framework, following Prasad (2024) [33]. GRADE offers a transparent, widely used method for rating confidence in findings and guiding evidence-informed decisions. Accordingly, all 42 studies (cross-sectional surveys, longitudinal cohorts, neuroimaging and qualitative interviews) were observational and therefore graded “low” certainty. Where serious concerns were identified, certainty ratings were downgraded by one level. Imprecision was considered serious when evidence was based on small samples, sparse data, unstable estimates, or confidence intervals that were wide or not reported. Inconsistency was considered serious when findings varied in direction or magnitude across studies and this variation could not be adequately explained by developmental stage, study design, measurement approach, or exposure characteristics. Indirectness was considered serious when the population, exposure, comparator, or outcome only partially corresponded to the review question, for example when studies relied on broader measures of screen time or social media use as proxies for short-form video exposure. One reviewer (M.E.) applied the initial GRADE ratings, which were then checked by a second reviewer (S.S.). All disagreements were resolved through discussion.

Conversely, no study met GRADE criteria for upgrading, such as large, uncontested effects, and therefore none reached “moderate” or “high” certainty. Thus, 12 studies retained “low” certainty, typically those with sizeable, demographically relevant samples, validated instruments, and no major flaws. In contrast, 30 studies were rated “very low” due to small or selective samples, inconsistent results, indirect measures, or imprecision. The complete rating matrix with domain-specific justifications and narrative notes is presented in Supplementary Material 4 on the OSF https://osf.io/qks32/files/osfstorage/684fc3904928e3899ab78802.

## Results

### General characteristics of the included studies

The electronic search yielded 1,842 records, of which 42 satisfied all pre-specified inclusion criteria and were therefore incorporated into the synthesis. All articles were published between 2021 and 2025. Most adopted cross-sectional quantitative designs (88%), two were longitudinal surveys [34, 35], one combined longitudinal and cross-sectional econometric methods [35], one used an experimental EEG paradigm [36], and one employed multimodal MRI [37]. The single qualitative study interviewed 7 participants [38]. Quantitative samples ranged from 29 [36] to 3,036 [39] (cumulative N = 29,600; mean study-mean age 16.8 years). Twenty-eight studies (67%) recruited adolescents (10–18 years), 13 focused on students 18–25 years of age, and one systematic review mixed both cohorts [32]. Excluding the international review, China accounted for 31 studies (76%), followed by Europe (6) and the Middle East (4). TikTok/Douyin was the sole platform examined in 24 papers; others referred to ’short-video platforms’ generally. Lastly, there was marked measurement heterogeneity (15 instruments for problematic SFV use or addiction), and no study used objective usage data (e.g., device logs or in-app analytics), instead relying on self-report. Moreover, there was no single definition of what ’excessive’ use is. The definitions ranged from more than 2 h/day [40] to more than 7 h/day [41], whereby two studies saw changes at >4 h [36, 42].

We grounded our analysis in Engel’s (1977) seminal biopsychosocial framework [23] to provide a well-established conceptual structure and improve interpretative rigour. In addition, an overview of the general characteristics is provided in Supplementary material S4. Those characteristics are: i) study design, ii) number and gender distribution of the participants, iii) age of the participants, iv) setting of the recruitment (e.g., school), v) country the study was conducted, vi) specific platform investigated, vii) assessment tools, viii) assessment outcome, ix) key findings, x) limitation, xi) GRADE quality rating. Supplementary material S4 is accessible for further use and meta-analysis by third parties via the OSF under: https://osf.io/qks32/files/osfstorage/684fc394cfe1cfc60fb786f2.

### Synthesis of major themes

#### Biological correlates

##### Molecular and genetic proxies

A neuroimaging-transcriptomic study of 111 Chinese college students reported associations between higher short-video addiction (SVA) scores and MRI-derived differences in grey-matter volume in the orbitofrontal cortex and cerebellum [37]. In this study, regions showing SVA-related grey-matter differences were subsequently examined in relation to normative brain gene-expression patterns. The transcriptomic component of the study did not involve direct genetic measurement in participants. Instead, the authors compared the spatial pattern of MRI findings with a reference atlas of brain gene expression.

This analysis identified 521 genes whose normative spatial expression patterns were associated with the short-video-addiction-related grey-matter map, including 288 positive and 233 negative correlations [37]. The strongest positive associations included KCNA5, WDR66, and ZMYND10, whereas the strongest negative associations included XKR9, SEMA7A, and RFPL2 [37]. However, because the analysis was atlas-based, cross-sectional, and correlational, it does not establish that these genes are altered in participants, nor that they contribute causally to short-video addiction. Accordingly, these molecular findings should be treated as hypothesis-generating rather than mechanistic evidence.

##### Neural circuits and electrophysiology

The same study [37] also reported associations with resting-state regional homogeneity in the dorsolateral prefrontal cortex, posterior cingulate cortex, cerebellum, and temporal pole. Regional homogeneity reflects the local synchronisation of spontaneous resting-state blood-oxygen-level-dependent (BOLD) signal fluctuations. Since the BOLD signal indexes change in blood oxygenation rather than neuronal firing directly, and since the haemodynamic response unfolds over several seconds, these findings should be interpreted as indirect markers of local resting-state signal synchrony rather than direct evidence of increased or decreased neural activity [37].

In a separate EEG study of 29 Austrian university students, participants completed a visual oddball task in which they discriminated rare white-circle targets from frequent white-triangle non-targets, and heavy short-video users showed a significantly smaller P300 amplitude than regular users (p = 0.013) [36]. Participants were classified as heavy users when they self-reported average SFV use above 4 hours per day. The study then analysed 14 heavy users and 15 regular users, with regular users defined as below 3 hours per day [36]. The P300 is an EEG response that appears about 300 milliseconds after a rare or unexpected stimulus, so a smaller P300 is usually taken to mean weaker attention-related processing or reduced allocation of attentional resources. While reduced P300 amplitude may indicate altered allocation of attentional resources, this interpretation remains tentative because of the small sample size, self-classification of user groups, and task-specific nature of event-related potential findings.

##### Working-memory capacity

two school-based studies linked heavier short-video use to poorer working memory and scholastic performance. In a Chinese study with 3,036 secondary-school students [39], higher TikTok-Use-Disorder scores predicted shorter forward- and backward-digit spans (total effects = -0.78, p < 0.001; -0.75, p < 0.001). The association was partly mediated by anxiety and depression, and, for the backward span, also by stress (p < 0.01). In a two-school sample [43] (N = 822), heavier daily viewing (r = 0.21, p < 0.05) combined with reliance on the personalised algorithmic feed (r = -0.15, p < 0.01) reduced working-memory span and verbal-reasoning scores, thus lowering GPA. The decrements were largest in boys and in the younger (12–13 years) cohort.

#### Biopsychological interactions

##### Anxiety

Firstly, anxiety consistently emerged as a potential intensifying factor in problematic SFV use and related psychosocial outcomes. In one study [39] higher TikTok-Use-Disorder scores predicted greater trait anxiety in Chinese secondary-school pupils (N = 3,036). Additionally, anxiety together with inattention, partly explained why excessive viewing increased school-refusal behaviour in a provincial survey of 2,056 adolescents [41]. A Greek online study of late adolescents [44] replicated the link (*β * = 0.404, p < 0.01), while structural modelling work in 804 Chinese undergraduates [45] showed that neuroticism raised anxiety, which then fed a depression-to-addiction pathway. Because each study captured only one time point, the direction of influence between anxiety and platform use remains open.

##### Attention

Secondly, attentional resources appeared to drain as SFV usage rose and impulsive traits magnified that loss. In a laboratory oddball paradigm [37], Austrian students who scrolled more than four hours a day produced a P300 wave smaller than that of regular users (p = 0.013), suggesting reduced neural capacity for novelty detection. Parallel school surveys offered behavioural corroboration: daily viewing predicted poorer attention and lower grade-point averages in 1,052 Chinese primary pupils (*β * = -0.25, p < 0.001) [46], while another Chinese study [41] recorded higher inattention scores alongside the anxiety noted above. Moreover, a cross-sectional survey [42] of 933 Egyptian undergraduates showed that watching “reels” emerged as an independent risk factor for positive ADHD screening (Exp B = 1.493, p < 0.01), whereas regular sport practice was reported as a protective factor (Exp B = 0.679, p = 0.016). Although no direct brain measure was taken, the finding aligns with attenuated attentional processing reported in the EEG study. Importantly, a structural-equation work [47] found two separate paths regarding how problematic SFV use occurred in 563 Chinese students: 1) inattention *→ * boredom *→ * problematic short-video use (PSVU), and 2) hyperactivity *→ * emotion distress *→ * boredom *→ * problematic use.

##### Depression

Thirdly, depression operated both as a hypothetical consequence of heavy SFV viewing and as a potential pathway into more intensive or problematic patterns of use. For instance, one Greek study [44] reported a depression coefficient of *β * = 0.472 (p < 0.001) after controlling for age and gender in a convenience sample of 219 adolescents. Another study with 1,302 Chinese senior high-schoolers [48] documented a dual pathway in which unmet entertainment and social needs, plus emergent addiction, channelled higher usage into depressive symptoms. Similarly, evidence from a two-wave Chinese study involving 480 participants [49], indicates that short-video addiction predicted subsequent depression. However this was observed only in adolescent boys. A broader systematic review [32] likewise concluded that intensive SFV exposure usually coincided with lower life satisfaction and higher depressive affect across ten countries. Nevertheless, the review did not investigate SFVs in isolation from more traditional (social) media [32].

##### Short-video addiction

Fourth, compulsive short-video viewing emerged as a highly reported mental health outcome. In online surveys of 659 Chinese adolescents [11], ’system quality’ and ’concentration’ together explained more problematic use than ’enjoyment’ alone. Structural equation modelling [50] showed that active parental mediation attenuated the flow-to-addiction path. A small (N = 96) exploratory poll [51] echoed the finding on system quality in finding that boredom and perceived recommender accuracy intensified compulsive scrolling. Interestingly, one study [52] found that among 1,047 under-graduates, self-rated short-video addiction predicted more frequent academic procrastination (*β * = 0.17, p < 0.001), while reduced attentional control partly bridged this link (r = -0.23, p < 0.001). Thereby, students low in boredom-proneness were paradoxically more at risk (*β * = 0.07, p < 0.05). Addiction, once entrenched, correlated with bedtime procrastination when self-control failed in adolescents [53] (N = 249, *β * = 0.532, p < 0.001) and university students [54] (N = 222, *β * = 0.259, p < 0.001), and with weaker self-regulated learning and learning well-being reported by Ye and colleagues with *β * = -0.40 (p < 0.01) in 2022 and *β * = −0.49 (p < 0.001) in 2024. Moreover, developmental work indicated that severe addiction rates doubled in a sample of 1,896 participants [35] from junior- to senior-high school and tripled to university with a peak at 52.7% “mild addiction” prevalence. This was driven in part by academic stress. Thereby, the authors used the following time-based cut-offs: “non-addicted” (< 1 h/day), “mild” (1-2 h/day), “moderate”, and “severe” (*≥ * 3 h/day). Additional antecedents included school burnout and social phobia [55], chronic loneliness [56], and single-country evidence [57] that daily stress can push adolescent boys (but not girls) into short-video addiction. Nevertheless, multiple factors might be mitigating such addiction spirals. Weekly physical exercise (*β * = -0.30, p < 0.01) reduced addiction scores in a 756 sample of adolescent participants [58] partly through enhancing self-control. Network analysis of 3,808 pupils [59] revealed that strong teacher-student ties curtailed symptoms in early adolescence, while peer connections took over this protective role in middle adolescence. In another study [60], future-focused temporal orientation and high dual-system self-control dampened addiction-risk in a 2,239 university student sample. Plus, questionnaire data [61] from the same setting (with N = 560) showed that proactive personality was found to lower addiction through resilience and top-down self-regulation. Qualitative interviews [38] also suggested that algorithm awareness and deliberate breaks helped university students prevent stress-relief scrolling from sliding into compulsion.

#### Psychological responses and individual vulnerabilities

##### Algorithmic resilience

First, a sense of agency vis-à-vis the recommendation feed appears to buffer young users. In Turkish high-schoolers [62], higher trait mindfulness indirectly enhanced psychological resilience through lowered problematic TikTok use and loneliness (*β * = 0.24, p < 0.001). Likewise, among 905 rural Chinese adolescents [63], perceiving the ‘For You’ interface as autonomy-threatening increased the intention to resist recommended clips (*β * = 0.21, t = 5.91), with the final model explaining 40% of behavioural variance (R$^{2}$ = 0.403; SRMR = 0.065). Yet, this protective profile faltered when two vulnerabilities coincided: parasocial idol bonds and FoMO. In Chinese university students [64], each intensified compulsive scrolling and TikTok fatigue. Paradoxically to the prior described findings, this was explained via greater algorithm awareness. The direct paths from parasocial bonds to awareness were non-significant, underscoring a fully mediated pattern (p < 0.05). The authors hypothesised that individuals with parasocial bonds and FoMO used their knowledge of how the algorithm works *to deepen* their engagement with these ‘vulnerabilities’, rather than algorithm awareness increasing resilience. Importantly, engagement style also mattered. Among 536 college students [65], social-support seeking translated into active posting (*β * = 0.60, p < 0.001), which fostered on-campus conversations (*β * = 0.35, p < 0.001) and, in turn, higher resilience (*β * = 0.15, p < 0.001). Conversely, passive swiping or chatting with strangers did not confer a resilience benefit. Nevertheless, all presented datasets for this subchapter are single-country, self-report, and cross-sectional, so reciprocal causation and cultural idiosyncrasies cannot be ruled out.

##### Self-regulation

Turning to behavioural self-control, two hours of daily TikTok use marked a behavioural tipping-point for Spanish adolescents [40]: each extra hour predicted poorer digital boundary-setting (p < 0.05), and girls both watched longer and consumed more beauty-and fashion content (p < 0.001). More fundamentally, the platform design variables seem to erode control by inducing flow. In a two-stage structural equation modelling (SEM) study [11] with 659 Chinese teens, system quality exerted a stronger total effect on flow experience than information quality. Flow then showed direct and indirect links to addiction, accounting for roughly one quarter of its variance (R$^{2}$ *≈ * 0.25). A related distinction separates exposure from impaired control. One study distinguished general TikTok use from TikTok self-control failure, defined as use that conflicts with valued goals or tasks [10]. Across middle-school students (N = 249) and university students (N = 222), self-control failure showed stronger associations with bedtime procrastination than duration or frequency. This pattern appeared in both adolescents (r = 0.532 vs. 0.275, p < 0.001) and university students (r = 0.259 vs. 0.055, p = 0.002). Self-control failure also related to poorer well-being in both samples (r = -0.290; r = -0.161). Among adolescents, it additionally correlated with poorer academic performance (r = -0.250). The authors argue that bedtime procrastination may be a more proximal outcome of self-control failure, whereas effects on well-being and grades may be more indirect. Protective dispositions and contexts counteracted that erosion. Among 560 Chinese undergraduates [61], proactive personality reduced addiction via self-control and resilience (total indirect *β * = -0.32, p < 0.001). Similarly, in a national sample of 1,735 middle-schoolers [66], a warm family climate curtailed addiction through two serial mediators: self-control alone (6.8% of the effect) and the chain from psychological-capital to self-control (12.8%). The zero-order family–addiction correlation was modest but highly significant (r = -0.19, p < 0.001). However, once addiction had set in, study skills shrank. Additional SEM work with 706 Chinese undergraduates [54] showed that addiction lowered self-regulated learning strategies, which in turn diminished learning well-being (all p < 0.05). Notably, all studies relied on self-rated control rather than app-log data, and most sampled a single city or university.

##### Stress loops

Stress proved to be an ubiquitous driver of compulsive short-video usage. School burnout (*β* *≈ * 0.29) and social phobia (*β* *≈ * 0.34) increased short-video addiction, which subsequently reduced happiness (*β* *≈ * -0.25) and parent-child relationship quality (*β* *≈ * -0.30) [55]. Furthermore, in 596 Chinese high-schoolers [57], perceived stress raised every smartphone-addiction subtype, in which short-video addiction associated with *β * = 0.32 (p < 0.001). Thereby, a stress-gender interaction (*β * = -0.39, p < 0.001) indicated a steeper pathway for boys. Qualitative interviews with UK students [38] echoed this loop. Initial stress relief quickly gave way to compulsive overuse and mood volatility.

##### Body comparison and eating-disorder risk

Evidence for body-image harm is mixed but gender-sensitive. Cross-sectionally, Saudi TikTok users [67] scored higher on social comparison (Median = 33 vs. 28, p = 0.005) and lower on body-image satisfaction (Median = 64 vs. 67, p = 0.037). In 795 Chinese adolescents [68], short-video-platform intensity predicted body dissatisfaction both directly (*β * = 0.25, p < 0.001) and indirectly through body-image comparison (BIC) (*β * = 0.2, p < 0.001), with pathways stronger for girls than boys (interaction *β * = 0.12, p < 0.001). Conversely, a ten-month random-intercept cross-lagged panel of 229 Belgian girls [34] found no prospective effect of TikTok minutes on either internalisation of beauty ideals or body-image discrepancy (all p > 0.34). Finally, survey data from an Italian eating disorder clinic [69] revealed that 59% of patients felt TikTok lowered self-esteem and that diet-hashtag searching was over-represented among those reporting poorer self-esteem.

##### Anger

Lastly, empirical links between short-video usage and anger remain elusive. In 1,176 Turkish teenagers [70], TikTok duration showed no association with anger once usage motives were controlled for. High general Instagram time related modestly to greater loneliness and Twitter use predicted higher anger. Therefore, the very limited evidence on this topic suggested that anger may stem more from platform motives than from scrolling SFVs per se.

#### Psychosocial patterns in personality, loneliness, and comparison

##### Bullying & peer friction

In a survey of 2,056 Chinese pupils [41], 31.9% (p = 0.005) met criteria for school refusal behaviour. Weekend excess in short-video viewing predicted school-refusal directly and indirectly through two mediators: inattention (standardised indirect *β * = 0.385) and school anxiety (*β * = 0.313) — with inattention carrying the larger share of the indirect effect. The paper reports full standardised coefficients and bootstrapped 95% confidence intervals, so the effect sizes are interpretable without additional caveats. When weekend short-video screen time exceeded 7 h per day, self-reported experiences of being bullied reached their highest levels. However, only the covert-bullying item remained a significant predictor of school-refusal in the random-forest analysis. The authors suggest this may reflect reduced face-to-face peer contact and weaker support networks caused by prolonged online viewing.

##### Engagement style (active vs. passive SFV usage)

System-wide patterns mirror this mechanistic picture. A 20-study review [32] reported that some of the primary studies associated passive watching with lower mental health indicators, among others, depression and stress. Yet, the conclusions of those 18 studies, which were cross-sectional and often did not isolate short-video platforms from other media, remain provisional. Interestingly, one of the stronger primary studies [65] replicated the nuance in engagement styles: among 536 Chinese undergraduates, support-seeking strongly promoted Douyin-updating (*β * = 0.60, p < 0.001), and on-campus friends modestly but significantly enhanced resilience (*β * = 0.15, p < 0.001).

##### Loneliness

Turning to loneliness, evidence is again bifurcated by context. Among 354 Turkish teens [62], higher dispositional mindfulness lowered problematic TikTok use and loneliness, together conveying a medium to large indirect boost to resilience (*β * = 0.24, p < 0.001). Conversely, in a study of 388 Chinese university students [56], loneliness emerged as a predictor of short-video addiction (*β * = 0.489, p < 0.001), with lower social support and reduced physical activity partially mediating this link. A third, platform-comparative study of 1,176 Turkish adolescents [70] found that heavy general Instagram, though not TikTok, use related to greater loneliness (p < 0.05).

##### Personality traits

Personality traits systematically alter the balance of power. In 804 Chinese undergraduates [45], high neuroticism and low agreeableness funnelled students into addiction via a depression to anxiety chain (all paths p < 0.001). By contrast, proactive personality exerted a protective total indirect effect in another study [61] (*β * = -0.320, p < 0.001) chiefly through enhanced self-control (*β * = -0.217, p < 0.001) and to a lesser degree through resilience (*β * = -0.063, p < 0.01). Clinical traits mattered too: among 563 students [47], ADHD-related inattention and hyperactivity-impulsivity displayed distinct indirect paths to problematic short-video usage (Estimate = 0.055 and 0.088 respectively, all p < 0.05), operating through boredom proneness, emotional distress and deficient reappraisal. Although many coefficients are modest, they converge on a pattern, whereby negative affectivity and low self-regulation propel overuse, whereas goal-directed agency counteracts it.

##### Self-concept clarity

Self-definition also showed susceptibility. In a sample of 721 early adolescents [71], greater short-video intensity predicted weaker self-concept clarity, indirectly by two routes: (i) via heightened flow (*β * = -0.12; p = 0.05) and (ii) via the serial path flow to online selfexpansion (*β * = -0.08; p = 0.05). These effects, though medium in size, are cross-sectional. Therefore, the direction could plausibly be reversed with identity-diffused teens having sought flow to escape uncertainty.

##### Social comparison

Finally, motives of use appeared pivotal when social comparison entered the frame. Reflexive thematic interviews with UK students [38] revealed that algorithm-pushed trauma disclosures and “perfect-body” clips triggered upward comparison and binge-watching cycles. Complementing this, a Chinese school survey [72] (N = 786) found, that ability- and opinion-based comparison did not directly impact flourishing. Instead, integration of short-videos into daily routines produced a small positive indirect effect, whereas emotional dependency exerted a small negative one (model fit: $\chi ^2$/df = 2.10 < 3, comparative fit index = 0.92 > 0.90, Tucker−Lewis index = 0.90 *≥ * 0.90, and RMSEA = 057 < 0.06).

#### Social environments and moderators

##### Virtual feed and curriculum

A two-cohort path analysis of 822 Chinese pupils [43] showed that each extra hour of daily short-video viewing coincided with lower delay-of-gratification scores (*β * = -0.45; p = 0.001) and poorer term grades (*β * = -0.33; p = 0.001) in both age groups, whereas algorithm-dependence additionally predicted weaker working-memory (*β * = -0.15; p = 0.01) and verbal-ability (*β * = -0.11; p = 0.05) and thereby even steeper grade loss in 12- to 13-year-olds. Similarly, a survey of senior pupils [46] linked heavier viewing to attentional drains and lower academic performance (*β * = -0.043, p = 0.000). Two convenience surveys of vocational and university students [53, 54] found that higher addiction scores undermined planning, monitoring and intrinsic learning motivation, collectively eroding study well-being (study 1: *β * = −0.40, p < 0.01 and study 2: *β * = −0.49, p < 0.001). Because both samples were self-selected, the absolute effect sizes remain uncertain, yet the pattern aligns with evidence from younger cohorts that attentional resources might be the critical bottleneck. When short-video binges collide with homework or bedtime, behavioural costs become more visible. A study with 1,047 undergraduates [52] documented a direct link from SV addiction to academic procrastination (*β * = 0.17, p < 0.001) and attentional control (r = -0.23, p < 0.001). Hereby, the academic procrastination was partly transmitted by attentional control (indirect path *β* *≈ * -0.34, p < 0.001), and the mediation was strongest among boredom-prone students (interaction *β * = 0.07, p < 0.05). At the school level, a provincial survey of 2,056 adolescents [41] reported that weekend viewing beyond seven hours raised school-refusal prevalence to 31.9%, with inattention and school anxiety jointly mediating the effect.

##### Parent−child dynamics

Household climate appeared to be crucial. In a SEM of 633 adolescents [50], the concentration stage of flow was the strongest predictor of problematic TikTok use, but active parental discussion significantly dampened this path, reducing the overall variance extracted (AVE = 0.641). Earlier work with 1,735 middle-schoolers [66] echoed the buffer: a supportive family environment curtailed addiction through a self-control pathway that explained 12.8% of the total effect. Conversely, when parents themselves bingescrolled, children’s attentional losses were higher (*β * = -0.092, p < 0.001) than solely the children’s time on SFVs (*β * = -0.071, p < 0.001) [46].

##### Other protective relationships

Turning to the wider school ecology, a large symptom-network study [59] (N = 3,808) showed that teacher-student relationship acted as the primary bridge away from addiction symptoms in primary school, whereas peer-closeness took over that role in Grade 8. In both networks the central nodes were “hard to control” and “increasing time for satisfaction”. Qualitative interviews with UK students [38] revealed that mental-health storytelling on TikTok can provide momentary relief yet morph into upward comparison and renewed anxiety with prolonged exposure. Moreover, quantitative data aligns. One study [64] found university students who formed parasocial bonds and experienced higher FoMO were not only more compulsive scrollers but also reported fatigue.

In contrast, a school survey [72] found that when short-videos were casually woven into daily routines in a goal-orientated way (e.g., ’how-to’ clips), comparison was linked to greater flourishing, whereas emotional attachment reversed the effect, predicting increased anxiety. Hence, *why* adolescents watch appears to be as critical as *how* they are invested and engage with SFVs. Finally, two independent surveys [42, 56] indicated that regular participation in team or club sports is associated with a lower risk of addiction, apparently due to reduced loneliness or inattention. Both samples were convenience-based and did not differentiate between organised and informal sports.

## Discussion

### Discussion of findings

This review provides a map that cross-walks observed outcomes onto biopsychosocial domains and annotates each domain with GRADE-informed certainty cues. The synthesised evidence reveals convergent neurocognitive findings centred on attention and reward circuitry, consistent with small-to-moderate neurocognitive costs. These findings are in strong alignment with the recent meta-analysis by Nguyen et al. (2025) [73], which offers a rigorous quantification of short-form video effects and an empirical benchmark for the field. Our youth-focused synthesis complements this work by narrowing the developmental window and by reporting model-based estimates from individual studies, which may produce larger coefficients than meta-analytic effect sizes. Together, the two reviews build and replicate the overarching effect structure while the present review refines how these associations manifest in younger users and highlights candidate factors that may be relevant for prevention and intervention.

Mechanistically, available evidence points to the reward and attention systems. One MRI study [37] reports an association between higher short-video addiction scores and orbitofrontal–cerebellar hypertrophy and co-expression of >500 dopaminergic and synaptic genes, while a small EEG experiment [36] (N = 29) detects a blunted P300 “novelty alarm” in daily four-hour-plus scrollers, which may reflect cue-driven, stimulus-bound behaviour. Although cross-sectional, these findings are consistent with rodent models of early-life audiovisual overstimulation that yield lasting disinhibition and cortical reorganisation mirroring ADHD-like features (see Section “Introduction”).

Cognitively, adolescents show shorter forward and backward digit spans with higher TikTok-Use-Disorder scores, partly channelled through anxiety, depression and stress [39]. Moreover, reliance on personalised feeds (e.g., “For You”) diminishes working-memory span and verbal reasoning, lowering GPA [43]. This association is especially pronounced in boys and in the youngest (12–13-year-old) cohort.

Psychologically, negative affect both precedes and follows heavy use. Elevated anxiety is associated with higher short-video use [44]. However, depressive symptoms follow addiction rather than precede it among adolescent boys [39]. Path models repeatedly identify three factors consistently associated with the maintenance of negative affect: boredom [47, 51, 52], flow-driven time distortion [11, 50, 71], and weak self-control [10, 40, 59]. In contrast, supportive parenting [46, 50, 65], regular exercise [58], algorithm literacy [43, 63, 64], and close teacher/peer ties [63] attenuate the link between heavy use and distress.

Limited longitudinal evidence from screen time research points to bidirectionality, whereby pre-existing ADHD symptoms can draw some young people to scroll SFVs excessively, and sustained use exacerbates those symptoms [16]. Set against pre-SFV syntheses – small associations with ADHD-related behaviour (*r=0.12*) and emphasis on temperament and gender over exposure volume [14, 15] – our SFV-specific synthesis suggests a modest upward shift with heavy use tied to a small-to-moderate rise in inattention and a moderate-to-large reduction in working-memory span.

Relationship scaffolds shift with age. Teacher–student bonds buffer compulsive use in primary years, while peer closeness takes over in early adolescence [59]. Within families, active parental discussion markedly decreases the addiction pathway [50, 65]. In contrast parental binge-scrolling is associated with a higher likelihood of child inattention [46]. This finding is consistent with existing calls for positive parental role-modelling from media researchers [74, 75].

Across studies, ’excessive’ use was operationalized with different cut-offs and categories (including more than 2h/day [40], more than 5 h and 7 h [41], and 4h/day [36, 42]). When considered collectively, ’excessive’ use is best treated as a *broad* elevated-use zone, potentially around 4 h/day, rather than a single universal cut-off.

#### Implications for prevention, diagnostics and intervention

This review offers a clinically usable framework for problematic SVF exposure by shifting attention from *what* is watched to *how* it is delivered, experienced, and regulated. On this basis, we translate the review findings into a practical overview of candidate levers for clinical and public health use (see Table 3). Although the evidence remains uncertain, several candidate moderators recur across studies, including algorithm literacy, self-regulatory capacity, structured digital routines, and supportive social environments. These variables should not be interpreted as deterministic “effects”. Rather, they represent plausible targets for future prevention and intervention research. In particular, approaches that increase users’ awareness of algorithmic curation and support more intentional engagement may be relevant across developmental stages, although empirical validation remains limited.

**Table 3:** Candidate levers associated with more favourable short-form video (SFV) use patterns across developmental stages

Candidate Levers	Definition	Evidence Strength	Developmental Stage	Example Implications
Digital routines and habits [52, 60, 72]	Time-based patterns of platform use and the extent to which engagement is deliberate versus automatic	Very Low–Low	Adolescents, young adults	Encourage intentional, goal-directed use. Set time boundaries, especially at night
Algorithm literacy [34, 43, 64, 67–69]	Awareness that feeds are algorithmically curated, shaping content exposure (including body-ideal content)	Very Low–Low	Adolescents, young adults	Educate on recommender systems and appearance-based curation. Foster critical feed engagement
Self-regulation/Self-control [10, 40, 52, 58–61, 66]	Capacity for impulse control, attention, delayed gratification, and disengagement from stimuli	Very Low–Low	Adolescents, young adults	Build self-regulatory skills. Support boundary setting beyond screen-time reduction
Parental mediation/Family environment [46, 50, 66]	Parental modelling, communication, and guidance shaping media norms	Very Low–Low	Childhood, early adolescence	Encourage active, dialogic mediation. Model balanced media use
Physical activity [42, 56, 58]	Regular exercise supporting cognitive, affective, and behavioural regulation	Very Low–Low	Adolescents, young adults	Integrate activity into daily routines to support self-regulation
Peer and teacher relationships [59]	Social support and connectedness in school settings	Low	Teacher influence in childhood. Peer influence in adolescence	Foster supportive teacher–student relationships and positive peer environments
Stress management and coping skills [35, 38, 55, 57]	Capacity to regulate daily stressors that can trigger or reinforce compulsive SFV use	Very Low–Low	Adolescents	Teach healthy coping alternatives to stress-relief scrolling.

Interestingly, the current scale literature overlaps with several of our candidate levers. Sleep-related items are most explicit in the Short-Video Dependence Scale (SVDS) [76], which includes prompts on staying up late and resisting short videos when trying to go to bed early. The Short Video Addiction Scale for Middle School Students (SVAS-MSS) [77] captures academic procrastination and time displacement, while the TikTok Addiction Scale (TTAS) [78] includes an item on considering reductions in work or study time to spend more time on TikTok. However, sleep-specific formulation appears less prominently in the TTAS and SVAS-MSS than in the SVDS. Bedtime procrastination therefore does not yet appear to be operationalised consistently across the scale literature. In the SVAS-MSS, the closest proxy is the time-displacement and procrastination cluster, rather than a direct sleep-related construct [77].

At the same time, algorithm literacy and perceived agency over the feed are largely absent from existing scales. This omission is notable because algorithmic personalisation is repeatedly discussed as a plausible mechanism contributing to problematic short-form video use. Current measures predominantly assess salience, loss of control, time displacement, and relational consequences, but do not directly capture whether users recognise, understand, or attribute their exposure to recommender systems [78, 79]. Consequently, future scale development should consider items that assess perceived algorithmic agency, for example, “Do you feel that you choose most of the videos you watch, or does the app mostly decide for you through its ‘For You’ page?”.

Family context is also represented only indirectly, mainly in the SVAS-MSS [77], through interpersonal strain and social communication difficulties involving parents, friends, and classmates. These items are useful for capturing relational consequences, but they primarily assess conflict after problematic use has emerged, rather than family environment as such. As a result, they do not yet measure constructs such as family warmth, parental monitoring, parental mediation, or broader household routines. This omission may be particularly relevant for children and early adolescents, as adjacent behavioural-addiction literature treats parent-child connectedness and parental mediation as meaningful protective or risk-relevant factors in the development of some forms of media addiction, rather than merely as sources of conflict [80].

### Limitations

One limitation of this review relates to the underlying database. China accounts for 76% of the available evidence, and 88% of the studies employ cross-sectional surveys. Such a geographically concentrated and cross-sectional corpus identifies associations but provides limited basis for causal inference regarding anxiety, ADHD-like symptoms, or compulsive short-video use, and it offers no basis for assessing international generalisability. A further limitation is that heavy, severe, or excessive use was not defined uniformly across studies, which makes direct comparison across findings harder and means that any single threshold should be treated as study-specific rather than field-wide. Existing laboratory experiments enrol very small samples, and none incorporate objective exposure metrics (e.g., device logs or in-app analytics).

Additional bias derives from the search strategy. The present review deliberately oversampled neuro-cognitive and negative-valence keywords. Terms such as ‘depression’ greatly exceeded positive constructs such as ‘flourishing’. Nonetheless, the corpus includes a null finding for anger and a positive association with flourishing, indicating that the strategy did not exclude all neutral or beneficial outcomes. Limiting the search to English- and German-language publications may have excluded region-specific studies published in other languages. Furthermore, because we replaced a full snowball strategy with only on review [32] as the primary citation benchmark, the resulting corpus of 42 studies is unlikely to be exhaustive. In addition, measurement heterogeneity complicated the synthesis. The researchers in the present review deployed no fewer than 15 different addiction scales, which diminishes the precision of the pooled estimate on short-video addiction.

The developmental interpretation of this review is limited by the scarcity of childhood studies, which may partly reflect platform age restrictions and the ethical and practical difficulties of studying potentially harmful digital exposures in younger children. Available childhood studies also often lack precise age reporting, referring broadly to “primary school students” rather than providing exact age ranges. Future childhood media research should routinely assess short-form video use and distinguish it from other activities occurring on the same platforms. For example, YouTube use should differentiate watching Shorts from watching long-form videos, while Instagram use should distinguish viewing Reels from messaging friends or browsing image-based posts. Without this distinction, platform-level measures may obscure the specific delivery mechanics of short-form video exposure, including algorithmic curation, infinite scroll, and rapidity.

Collectively, these constraints mean that the convergent patterns should be read as provisional signals rather than definitive causal evidence. The strengths of the present work lie elsewhere: this review isolates SFVs from gaming and legacy social media, foregrounds the role of their user interface design for neuroscience, and frames the evidence within a developmental biopsychosocial model to identify candidate levers for future research and practice.

## Conclusion

The currently available evidence is provisional but coherent. Although this evidence is methodologically limited (predominantly cross-sectional, China-centric, and self-reported), high exposure to SFVs is frequently associated with less favourable mental-health indicators among young people. Namely, the associations are: higher attentional difficulties, lower working-memory performance, higher anxiety and depression, weaker self-regulation, and addiction-like patterns. Moreover, novel EEG and MRI findings are consistent with the attention pathway, although mechanistic interpretations remain preliminary. However, effects are not uniform. Resilience-promoting factors (family climate, social ties at school, digital routines and algorithm literacy) are associated with variation in risk indicators, and some engagement styles (goal-directed, socially embedded engagement) appear to be associated with more favourable outcomes than passive consumption. Treating all screen media alike or recommending blanket bans may lead to poorly targeted guidance. The benefit of SFV-specific guidance is better-matched assessment and prevention.

Accordingly, rigorous longitudinal cohorts with objective exposure measurement (e.g., device/app logs) and trials of modifiable levers are urgently needed. Candidate levers include interface redesigns, algorithm-literacy modules in school, digital routine promotion, and dialogue-centred parental mediation.

Until new evidence accumulates, proportionate and transparent guidance may be appropriate: (i) equip children and adolescents with algorithm literacy and self-regulation skills, (ii) support parents to model balanced habits and encourage mediation, (iii) develop and validate measurement items that capture algorithm dependency within short-form video addiction scales, and (iv) maintain transparency about the field’s low-to-very-low certainty. This recognises both the creative potential of SFVs and the non-trivial risks of their design, aligning guidance with what matters most to practice and policy right now.

## Supplementary information

All supplementarys materials are available on the OSF.

**S1_syntax_protocol.xlsx:**

https://osf.io/qks32/files/osfstorage/68d0341f49bfe7549f53d908

**S2_full_text_suitability.xlsx:**

https://osf.io/qks32/files/osfstorage/684fc393567a560b45cc2c71

**S3_snowball_search.xlsx:**

https://osf.io/qks32/files/osfstorage/684fc38ce0b46fc2a7b7880c

**S4_GRADE.docx:**

https://osf.io/qks32/files/osfstorage/684fc3904928e3899ab78802

**S5_result_matrix.docx:**

https://osf.io/qks32/files/osfstorage/684fc394cfe1cfc60fb786f2

## Data Availability

No datasets were generated or analysed during the current study.
